# Supplementary material for: An At-Home Laparoscopic Curriculum for Junior Residents in Surgery, Obstetrics/Gynecology, and Urology
Source: MedEdPORTAL. 2024 May 24;20:11405. doi: 10.15766/mep_2374-8265.11405 (PMC11219092; doi:10.15766/mep_2374-8265.11405)
Supplement: Supplementary file 1 — At-Home Task Examples.mp4At-Home Task Descriptions and Rubrics.docxEquipment.docxEnd-of-Curriculum Assessment Overview.docxAssessment Task Descriptions and Rubrics.docxAssessment Station Examples.mp4 [file mep_2374-8265.11405-s001.zip › D. End-of-Curriculum Assessment Overview.docx]

**End-of-Curriculum Assessment Overview**

This appendix describes the end-of-curriculum assessment, including its objectives, preparation, set up, debriefing, and clean up. Use this appendix to plan and execute the end-of-curriculum assessment.

*Note that all pictures are author created and owned and have not been publicly distributed previously.*

**Objectives:**

- Transfer basic laparoscopic skills from at-home curriculum and obtain feedback from expert faculty​
- Appreciate laparoscopic skill progression and skills gained​
- Demonstrate confidence in foundational skills to encourage authentic participation in the operating room

**Pre-Session:**

- Recruit faculty members who perform laparoscopy to attend, facilitate stations, and assess residents
- Purchase 18-inch-long cow small intestines from a local butcher
- Print a copy of the assessment form (Appendix E) for each participant

**Session Set Up:**

- Prepare six laparoscopic assessment stations
  - If six laparoscopic trainers are not available, stations one, two, and three can be combined
- Place a stopwatch and the relevant assessment forms at every station
- Specific set up by station:
- Station one - Enterotomy + Foreign Body Retrieval: Place marbles inside each length of intestine (one marble per resident). Arrange the intestine on a tray as below. Place the following instruments at the station:
  - Bowel graspers x2
  - Laparoscopic Scissors


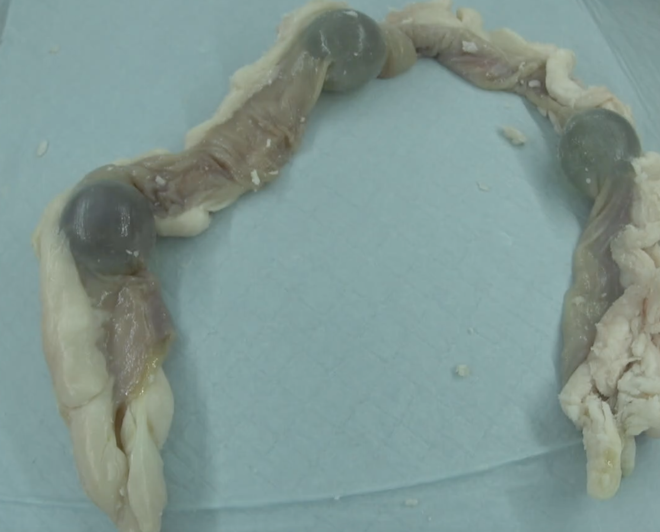


- Station two - Running the Bowel: Create an enterotomy in a length of intestine by snipping off part of the intestine along the antimesenteric border. Clip one end of the intestine to the edge of a tray with a medium binder clip, and coil the remaining intestine in the corner of the tray as below. Place the following instruments at the station:
  - Bowel graspers x2


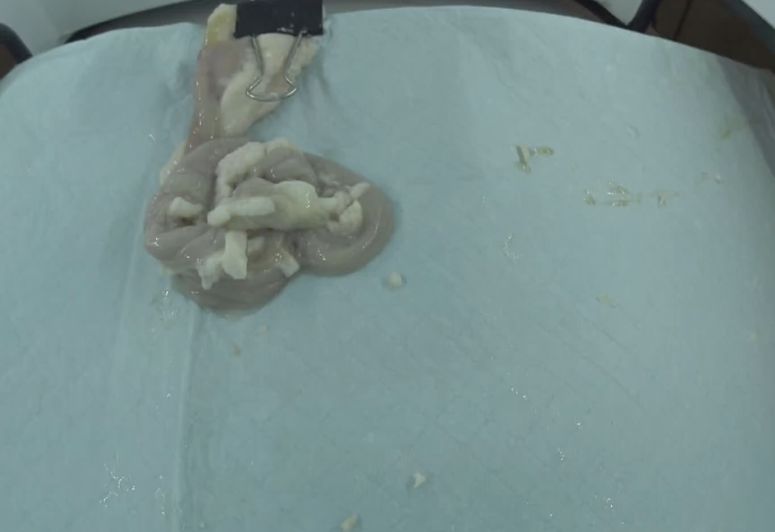


- Station three - Needle Loading: Place a length of intestine (or any soft material) on a tray, as below. The exact configuration of the intestine (or soft material) is unimportant. Place the following instruments/sutures at the station:
  - Laparoscopic needle driver x2
  - Laparoscopic Maryland x1
  - 3-0 silk (or any braided) suture with tail cut to 5cm x2


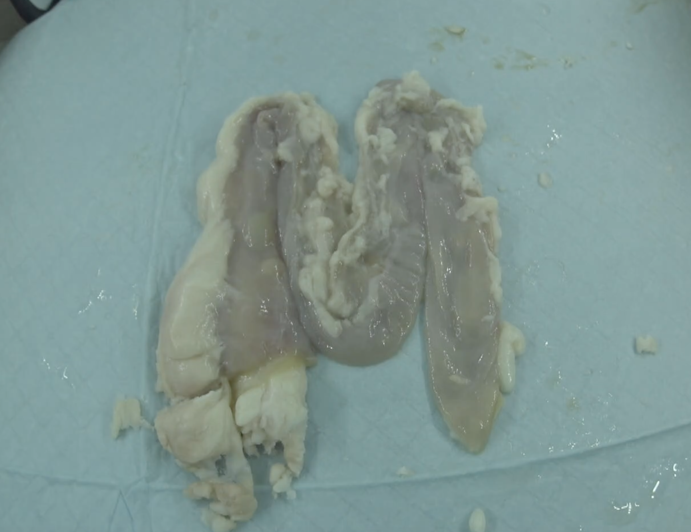


- Station four - Throwing Stitches: Place a length of intestine in an “M” shape on a tray as shown in the picture for station three. Place the following instruments/sutures at the station:
  - Laparoscopic needle driver x2
  - Laparoscopic Maryland x1
  - 3-0 silk (or any braided) suture with tail cut to 5cm
- Station five - Extracorporeal Suturing: Place a length of intestine in an “M” shape on a tray as shown in the picture for station three. Place the following instruments/sutures at the station:
  - Laparoscopic needle driver x2
  - Laparoscopic Maryland x1
  - Laparoscopic knot pusher
  - Laparoscopic Scissors
  - 3-0 silk (or any braided) suture with a length of 90 to 120cm
- Station six - Intracorporeal Suturing: Pre-cut a 3-0 silk (or any braided) suture to a length of 15cm and drive through two apposed segments of the length of intestine, as below. Do not pull the suture all the way through the intestine. Repeat for the number of residents who will participate. Place the following instruments/sutures at the station:
  - Laparoscopic needle driver x2
  - Laparoscopic Maryland x1
  - Laparoscopic Scissors


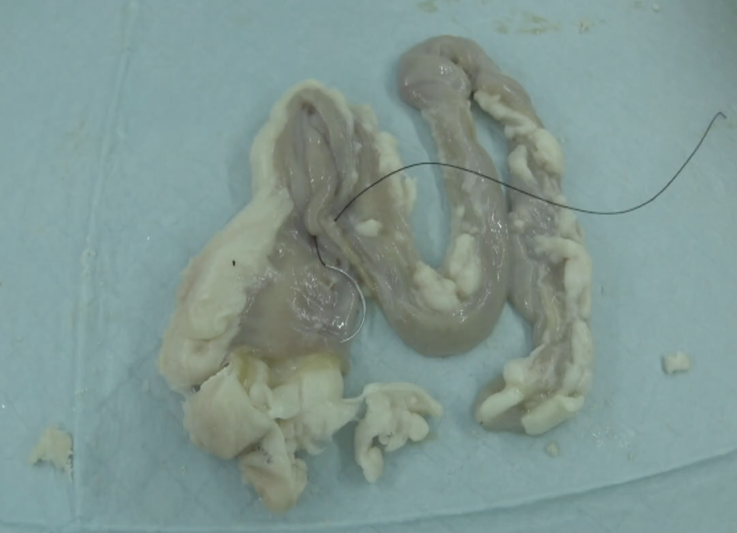


**Task Descriptions:**

Tasks descriptions and rubrics are available for each station in Appendix E.

**Debriefing:**

- After residents have completed all six stations, gather residents and faculty together to debrief
- Suggested debriefing topics include:
  - Discussing how at-home practice translating to operating on tissue
  - Describing barriers to performance and exchanging tips on overcoming barriers
  - Reviewing key strategies for challenging skills, such as loop formation and tail management
  - Emphasizing importance of ongoing skill development and discussing methods to continue asynchronous at-home practice

**Clean Up:**

- Before session ends, prepare bins for individual instruments
- Soak instruments in an enzymatic solution or soap
  - Open instruments in bin to allow soaking to reach all areas of instrument
- Rinse well, place instruments on paper towel lined trays to dry (all instruments should be opened)
- Discard bovine intestines
- Wipe down all tables and equipment touched by intestines, including floors, counter tops, etc.
