# Supplementary material for: An At-Home Laparoscopic Curriculum for Junior Residents in Surgery, Obstetrics/Gynecology, and Urology
Source: MedEdPORTAL. 2024 May 24;20:11405. doi: 10.15766/mep_2374-8265.11405 (PMC11219092; doi:10.15766/mep_2374-8265.11405)
Supplement: Supplementary file 1 — At-Home Task Examples.mp4At-Home Task Descriptions and Rubrics.docxEquipment.docxEnd-of-Curriculum Assessment Overview.docxAssessment Task Descriptions and Rubrics.docxAssessment Station Examples.mp4 [file mep_2374-8265.11405-s001.zip › B. At-Home Task Descriptions and Rubrics.docx]

| \| Laparoscopic Curriculum \| 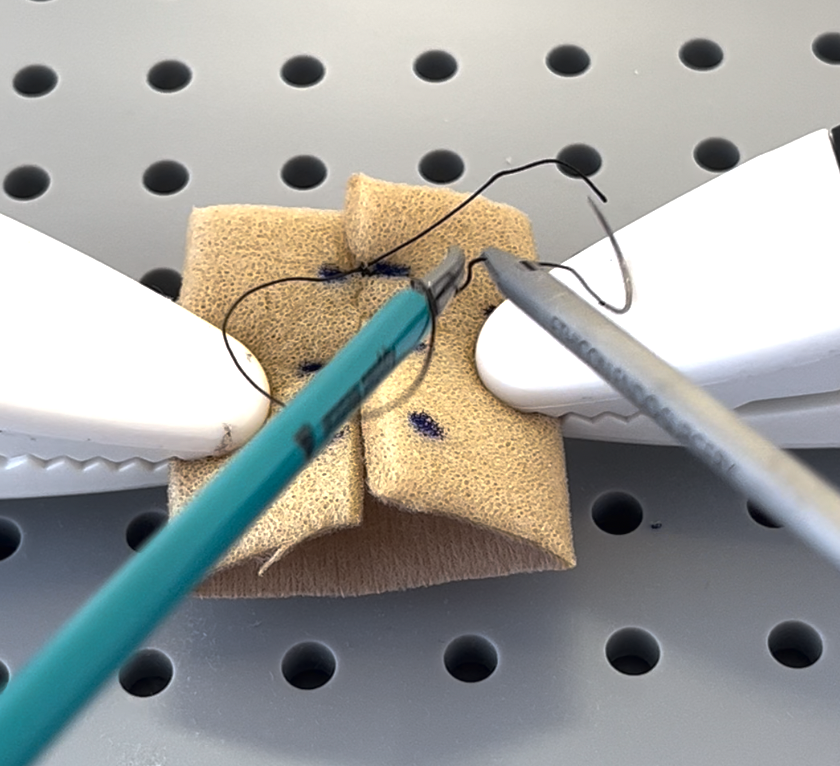 \| \| --- \| --- \| \| 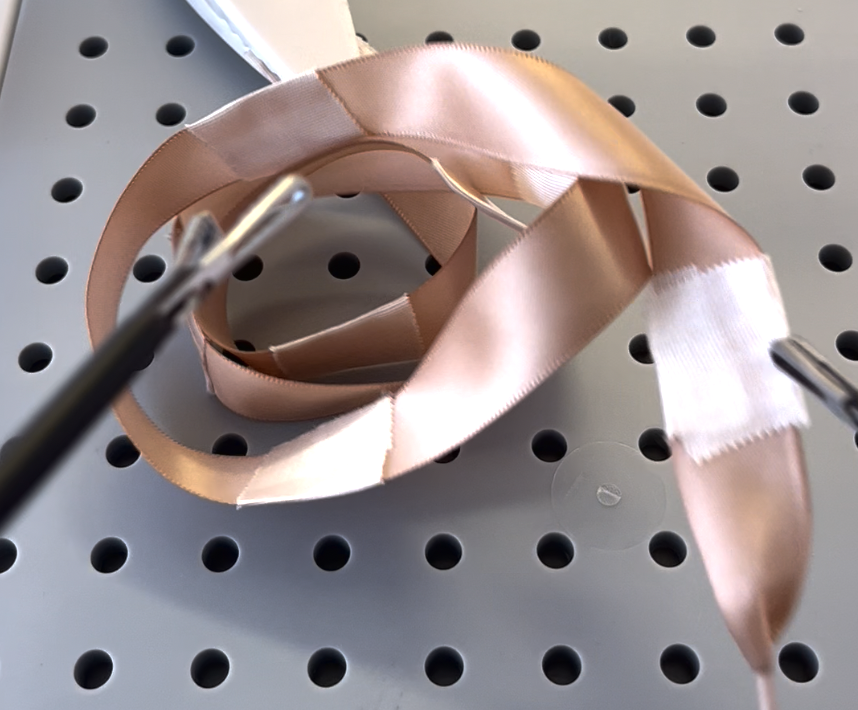 \| 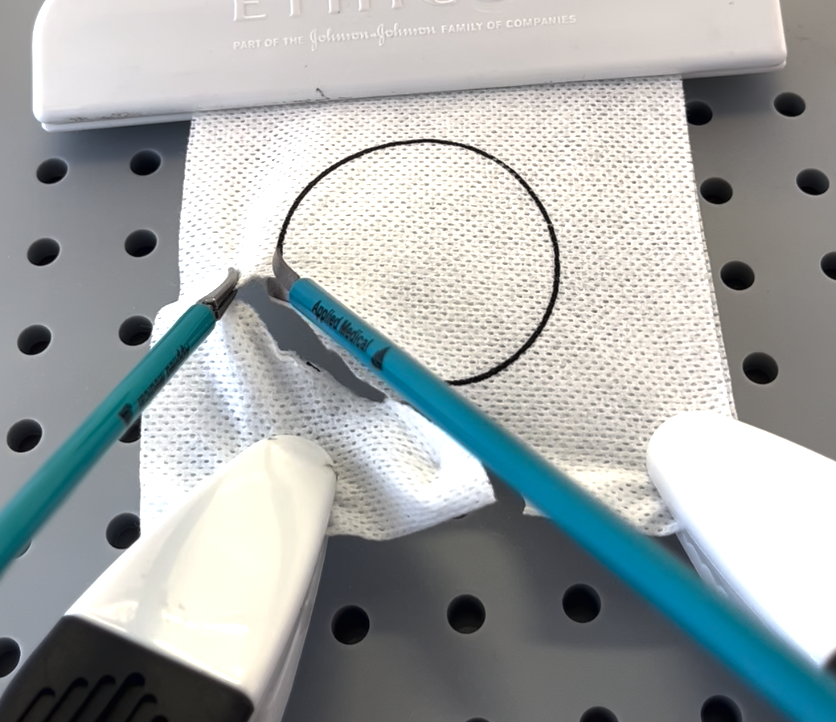 \| |
| --- | --- | --- | --- | --- |
| At-Home Task Descriptions and Rubrics  This manual includes information on performing and assessing the at-home laparoscopic tasks. Provide this manual to residents prior to starting the curriculum. Residents could spend five minutes to two hours practicing and recording each task.  Note that all pictures are author created and owned and have not been publicly distributed previously. |

# STAGE ONE: PEG TRANSFER

*Task description:* Use two Maryland graspers. Lift each object through its center hole with your non-dominant hand and transfer the object in midair to your dominant hand. Place the object onto the other side of the pegboard. This is then reversed and objects are transferred back to the original side of the pegboard. Aim to complete the task in 53 seconds or less.

- Time
- **4 points:** 53 seconds or less
- **3 points:** 54 to 80 seconds
- **2 points:** 81 to 96 seconds
- **1 point:** 97 seconds or more
- Hands
- **3 points:** Both hands are generally in motion to execute one transfer while preparing for the next
- **2 points:** Both hands are used in the task, but one is often static
- **1 point:** Only one hand is used in the task
- Visuospatial
- **2 points:** Optimal visualization is maintained throughout by performing transfers in the center of the screen
- **1 point:** Transfers for peripheral objects are performed in the edge of the field of vision
- Dexterity
- **3 points:** All objects are transferred between hands and placed on the pegs without being dropped
- **2 points:** One object is dropped during transfer and is picked up by the dropping instrument
- **1 point:** More than one object is dropped or a dropped object is picked up by the non-dropping instrument
- Support
- **2 points:** All objects are transferred in midair without the support of pegs or the board
- **1 point:** Transfers are performed by supporting transfers on the pegs or the board
- Instrumentation
- **2 points:** The instrument jaws are directed downward to grasp each object through its center hole
- **1 point:** The instrument jaws are directed upwards or grasp one or more objects from the side
- Collisions
- **2 points:** The laparoscopic instruments (Marylands) do not collide with each other
- **1 point:** Instrument arms collide at any point

# STAGE TWO: RUNNING THE “BOWEL”

*Task description:* Use two graspers. Place a clip in the upper outer part of the peg board and attach the end of the marked rope. Curl the rope in a tight circle next to the clip. Starting with the free end of the rope, grab the first and second marks on the rope and then grab each subsequent mark on the rope without touching the unmarked parts of the rope. Aim to complete the task in 28 seconds or less.

- Time
- **4 points:** 28 seconds or less
- **3 points:** 29 to 38 seconds
- **2 points:** 39 to 48 seconds
- **1 point:** 49 seconds or more
- Visuospatial
- **3 points:** Both instruments are kept in the field of view
- **2 points:** An instrument is moved out of the field of view while the jaws are empty
- **1 point:** An instrument is moved out of the field of view while grasping the rope
- Dexterity
- **3 points:** Only the marked parts of the rope are touched by the graspers
- **2 points:** The non-marked part of the rope is touched by a grasper once
- **1 point:** The non-marked part of the rope is touched by a grasper two or more times
- Handling
- **3 points:** The rope is not pulled out of or moved within the clip
- **2 points:** The rope is moved within the clip but not pulled out
- **1 point:** The rope is pulled out of the clip

# STAGE THREE: PRECISION CUTTING

*Task description:* Use any grasper in your nondominant hand and scissors in your dominant hand to cut a 4-cm diameter circular pattern out of a piece of gauze suspended between clips. Use the grasper to maintain the material under tension while cutting with the scissors in the other hand. Aim to complete the task in 50 seconds or less.

- Time
- **4 points:** 50 seconds or less
- **3 points:** 51 to 75 seconds
- **2 points:** 76 to 100 seconds
- **1 point:** 101 seconds or more
- Precision
- **4 points:** A circle is cut out from the gauze without entering the marked zone
- **3 points:** A circle is cut out from the gauze, but the marked zone is violated in one spot
- **2 points:** A circle is cut out from the gauze, but the marked zone is violated in two or more spots
- **1 point:** A circle is not cut out from the gauze
- Forward Planning
- **2 points:** The entry cut site is selected in a way that optimizes the alignment of the scissors with the pattern
- **1 point:** The entry cut site does not optimize the alignment of the scissors with the pattern
- Tension
- **3 points:** Appropriate tension is maintained with the nondominant hand by re-grabbing and triangulating
- **2 points:** Appropriate tension is occasionally maintained with the nondominant hand by re-grabbing; triangulation is infrequently performed
- **1 point:** The nondominant hand is not used to provide tension
- Instrumentation
- **2 points:** The orientation of the scissors is adjusted based on the pattern’s curvature
- **1 point:** The orientation of the scissors does not align with the pattern’s curvature

# STAGE FOUR: NEEDLE LOADING, PART 1

*Task description:* Use a needle driver in your dominant hand and a Maryland grasper in your nondominant hand. Introduce a needle with at least a 5cm tail onto the field with your Maryland grasper. Load the needle onto the needle driver in a forehand configuration. Aim to complete the task in 9 seconds or less.

- Time
- **4 points:** 9 seconds or less
- **3 points:** 10 to 14 seconds
- **2 points:** 15 to 19 seconds
- **1 point:** 20 seconds or more
- Instrumentation
- **3 points:** The Maryland grasper manipulates the needle to present it in the appropriate orientation for the needle driver without having to be reloaded
- **2 points:** The Maryland grasper manipulates the needle to present it in the appropriate orientation for the needle driver, but requires one or more reloadings
- **1 point:** The Maryland grasper does not present the needle in the appropriate orientation
- Dexterity
- **3 points:** The needle is transferred from the Maryland grasper to the needle driver and angled properly
- **2 points:** The needle is transferred from the Maryland grasper to the needle driver and angled in a suboptimal way
- **1 point:** The needle is not transferred from the Maryland grasper to the needle driver

# STAGE FIVE: NEEDLE LOADING, PART 2

*Task description:* Use a needle driver in your dominant hand and a Maryland grasper in your nondominant hand. Introduce a needle with at least a 5cm tail onto the field with your needle driver. Use the Maryland grasper to load the needle onto the needle driver in a forehand configuration. Aim to complete the task in 12 seconds or less.

- Time
- **4 points:** 12 seconds or less
- **3 points:** 13 to 19 seconds
- **2 points:** 20 to 26 seconds
- **1 point:** 27 seconds or more
- Instrumentation (1)
- **3 points:** The needle driver manipulates the needle to present it in the appropriate orientation for the Maryland grasper without having to be reloaded
- **2 points:** The needle driver manipulates the needle to present it in the appropriate orientation for the Maryland grasper, but requires one or more reloadings
- **1 point:** The needle driver does not present the needle in the appropriate orientation
- Instrumentation (2)
- **3 points:** The Maryland grasper manipulates the needle to present it in the appropriate orientation for the needle driver without having to be reloaded
- **2 points:** The Maryland grasper manipulates the needle to present it in the appropriate orientation for the needle driver, but requires one or more reloadings
- **1 point:** The Maryland grasper does not present the needle in the appropriate orientation
- Dexterity
- **3 points:** The needle is transferred from the Maryland grasper to the needle driver and angled properly
- **2 points:** The needle is transferred from the Maryland grasper to the needle driver and angled in a suboptimal way
- **1 point:** The needle is not transferred from the Maryland grasper to the needle driver

# STAGE SIX: SIMPLE INTERRUPTED EXTRACORPOREAL SUTURING

*Task description:* Use a needle driver in your dominant hand and a Maryland grasper or needle driver in your nondominant hand. Have scissors and a knot pusher nearby. Place the suture pad with the slit oriented vertically. Suture between two dots using a suture of 90 to 120cm in length. Tie three single throws extracorporeally and secure them with the knot pusher. Cut the ends. Aim to complete the task in 99 seconds or less.

- Time
- **4 points:** 99 seconds or less
- **3 points:** 100 to 149 seconds
- **2 points:** 150 to 199 seconds
- **1 point:** 200 seconds or more
- Precision
- **4 points:** The needle passes through the center of the marked holes
- **3 points:** The needle passes within the periphery of the marked holes
- **2 points:** The needle passes through part of one of the marked holes but not the other marked hole
- **1 point:** The needle does not pass through either marked hole
- Knot Security
- **3 points:** There is no air knot
- **2 points:** There is a small air knot, but the pad is still well approximated
- **1 point:** There is a significant air knot and the pad is not well approximated
- Handling
- **2 points:** The suture pad is not pulled off of the board
- **1 point:** The suture pad is pulled off the board
- Dexterity
- **3 points:** The needle is transferred from the Maryland grasper to the needle driver and angled properly
- **2 points:** The needle is transferred from the Maryland grasper to the needle driver and angled in a suboptimal way
- **1 point:** The needle is not transferred from the Maryland grasper to the needle driver
- Tension
- **3 points:** Appropriate tension is maintained with the nondominant hand by re-grabbing and triangulating the suture pad
- **2 points:** Appropriate tension is occasionally maintained with the nondominant hand by re-grabbing; triangulation is infrequently performed
- **1 point:** The nondominant hand is not used to provide tension

# STAGE SEVEN: SIMPLE INTERRUPTED INTRACORPOREAL SUTURING, PART 1

*Task description:* Use a needle driver in your dominant hand and a Maryland grasper or needle driver in your nondominant hand. Have scissors nearby. Place the suture pad with the slit oriented vertically. Suture between two dots using a suture of 15cm length. Tie a surgeon’s knot followed by two additional throws. You must exchange hands between each throw. Cut the ends. Aim to complete the task in 96 seconds or less.

- Time
- **4 points:** 96 seconds or less
- **3 points:** 97 to 160 seconds
- **2 points:** 161 to 244 seconds
- **1 point:** 245 seconds or more
- Precision
- **4 points:** The needle passes through the center of the marked holes
- **3 points:** The needle passes within the periphery of the marked holes
- **2 points:** The needle passes through part of one of the marked holes but not the other marked hole
- **1 point:** The needle does not pass through either marked hole
- Tail Management
- **3 points:** The tail end is kept sufficiently short to facilitate knot tying
- **2 points:** The tail end is too long but the tie is completed without creating a bow
- **1 point:** The tail end is too long and a bow is creating when tying
- Loop Management
- **3 points:** The two instruments move synchronously to form the loop and grab the tail end of the suture
- **2 points:** A loop is formed but only one instrument moves to grab the tail
- **1 point:** No loop is formed
- Knot Security
- **3 points:** There is no air knot
- **2 points:** There is a small air knot, but the pad is still well approximated
- **1 point:** There is a significant air knot and the pad is not well approximated
- Handling
- **2 points:** The suture pad is not pulled off of the board
- **1 point:** The suture pad is pulled off the board
- Dexterity
- **3 points:** The needle is transferred from the Maryland grasper to the needle driver and angled properly
- **2 points:** The needle is transferred from the Maryland grasper to the needle driver and angled in a suboptimal way
- **1 point:** The needle is not transferred from the Maryland grasper to the needle driver
- Tension
- **3 points:** Appropriate tension is maintained with the nondominant hand by re-grabbing and triangulating the suture pad
- **2 points:** Appropriate tension is occasionally maintained with the nondominant hand by re-grabbing; triangulation is infrequently performed
- **1 point:** The nondominant hand is not used to provide tension

# STAGE EIGHT: RUNNING INTRACORPOREAL SUTURING

*Task description:* Use a needle driver in your dominant hand and a Maryland grasper or needle driver in your nondominant hand. Place the suture pad with the slit oriented vertically. Start suturing using a suture of 15cm length between the two dots on the suture pad farthest from you. After this first pass, tie a surgeon’s knots with two additional throws. You must exchange hands between each throw. Run the stitch down to the bottom two dots. After passing the needle through the final two dots, no additional knot is required. Aim to complete the task in 165 seconds or less.

- Time
- **4 points:** 165 seconds or less
- **3 points:** 166 to 225 seconds
- **2 points:** 226 to 285 seconds
- **1 point:** 286 seconds or more
- Precision
- **4 points:** The needle passes through the center of the marked holes
- **3 points:** The needle passes within the periphery of the marked holes
- **2 points:** The needle passes through part of one of the marked holes but not the other marked hole
- **1 point:** The needle does not pass through either marked hole
- Tail Management
- **3 points:** The tail end is kept sufficiently short to facilitate knot tying
- **2 points:** The tail end is too long but the tie is completed without creating a bow
- **1 point:** The tail end is too long and a bow is creating when tying
- Loop Management
- **3 points:** The two instruments move synchronously to form the loop and grab the tail end of the suture
- **2 points:** A loop is formed but only one instrument moves to grab the tail
- **1 point:** No loop is formed
- Knot Security
- **3 points:** There is no air knot
- **2 points:** There is a small air knot, but the pad is still well approximated
- **1 point:** There is a significant air knot and the pad is not well approximated
- Handling
- **2 points:** The suture pad is not pulled off of the board
- **1 point:** The suture pad is pulled off the board
- Dexterity
- **3 points:** The needle is transferred from the Maryland grasper to the needle driver and angled properly
- **2 points:** The needle is transferred from the Maryland grasper to the needle driver and angled in a suboptimal way
- **1 point:** The needle is not transferred from the Maryland grasper to the needle driver
- Tension
- **3 points:** Appropriate tension is maintained with the nondominant hand by re-grabbing and triangulating the suture pad
- **2 points:** Appropriate tension is occasionally maintained with the nondominant hand by re-grabbing; triangulation is infrequently performed
- **1 point:** The nondominant hand is not used to provide tension

# STAGE NINE: SIMPLE INTERRUPTED INTRACORPOREAL SUTURING, PART 2

*Task description:* Use a needle driver in your dominant hand and a Maryland grasper or needle driver in your nondominant hand. Have scissors nearby. Place the suture pad with the slit oriented vertically. Start suturing between the two dots on the suture pad farthest from you using a suture of 18cm length. Tie a surgeon’s knot followed by two additional throws. You must exchange hands between each throw. Cut the ends. Repeat for the middle and lower dots on the suture pad. Aim to complete the task in 330 seconds or less.

- Time
- **4 points:** 330 seconds or less
- **3 points:** 331 to 519 seconds
- **2 points:** 520 to 708 seconds
- **1 point:** 709 seconds or more
- Precision
- **4 points:** The needle passes through the center of the marked holes in all throws
- **3 points:** The needle passes within the periphery of the marked holes in one or more throws
- **2 points:** The needle passes through part of one of the marked holes but not the other marked hole in one or more throws
- **1 point:** The needle does not pass through either marked hole in any throw
- Tail Management
- **3 points:** The tail end is kept sufficiently short to facilitate knot tying
- **2 points:** The tail end is too long but the tie is completed without creating a bow
- **1 point:** The tail end is too long and a bow is creating when tying
- Loop Management
- **3 points:** The two instruments move synchronously to form the loop and grab the tail end of the suture
- **2 points:** A loop is formed but only one instrument moves to grab the tail
- **1 point:** No loop is formed
- Knot Security
- **3 points:** There is no air knot
- **2 points:** There is a small air knot, but the pad is still well approximated
- **1 point:** There is a significant air knot and the pad is not well approximated
- Handling
- **2 points:** The suture pad is not pulled off of the board
- **1 point:** The suture pad is pulled off the board
- Dexterity
- **3 points:** The needle is transferred from the Maryland grasper to the needle driver and angled properly
- **2 points:** The needle is transferred from the Maryland grasper to the needle driver and angled in a suboptimal way
- **1 point:** The needle is not transferred from the Maryland grasper to the needle driver
- Tension
- **3 points:** Appropriate tension is maintained with the nondominant hand by re-grabbing and triangulating the suture pad
- **2 points:** Appropriate tension is occasionally maintained with the nondominant hand by re-grabbing; triangulation is infrequently performed
- **1 point:** The nondominant hand is not used to provide tension
