# Supplementary material for: An At-Home Laparoscopic Curriculum for Junior Residents in Surgery, Obstetrics/Gynecology, and Urology
Source: MedEdPORTAL. 2024 May 24;20:11405. doi: 10.15766/mep_2374-8265.11405 (PMC11219092; doi:10.15766/mep_2374-8265.11405)
Supplement: Supplementary file 1 — At-Home Task Examples.mp4At-Home Task Descriptions and Rubrics.docxEquipment.docxEnd-of-Curriculum Assessment Overview.docxAssessment Task Descriptions and Rubrics.docxAssessment Station Examples.mp4 [file mep_2374-8265.11405-s001.zip › C. Equipment.docx]

**Equipment**

This appendix details the equipment needed for the at-home curriculum and the end-of-curriculum assessment. Use this appendix to gather the necessary supplies. As described in the ESR Introduction, many options exist for obtaining these items, including industry-donated materials, purchased kits, 3D-printed supplies, and manually assembled materials.

At-home curriculum equipment:

- Collapsible training box
- Plastic training box floor
- Webcam or phone with camera
- One large white plastic clip
- Two small white plastic clips
- Gauze with circle pattern
- Peg board with six objects
- Foam pad
- Ribbon with taped intervals
- 3-0 silk (or any braided) sutures
- Laparoscopic instruments
  - Needle driver (1)
  - Maryland graspers (2)
  - D&G or bowel graspers (2)
  - Scissors (1)
  - Knot pusher (1)

End-of-curriculum assessment equipment:

- Laparoscopic trainers (4-6)
- Bovine intestine cut to 18 inches (may be frozen then thawed)
- Marbles
- Medium binder clip
- Bowel graspers (4)
- Laparoscopic Scissors (3)
- Laparoscopic needle driver (8)
- Laparoscopic Maryland (4)
- Laparoscopic knot pusher
- 3-0 silk (or any braided) suture with tail cut to 5cm (3)
- 3-0 silk (or any braided) suture with a length of 90 to 120cm
