# Supplementary material for: An At-Home Laparoscopic Curriculum for Junior Residents in Surgery, Obstetrics/Gynecology, and Urology
Source: MedEdPORTAL. 2024 May 24;20:11405. doi: 10.15766/mep_2374-8265.11405 (PMC11219092; doi:10.15766/mep_2374-8265.11405)
Supplement: Supplementary file 1 — At-Home Task Examples.mp4At-Home Task Descriptions and Rubrics.docxEquipment.docxEnd-of-Curriculum Assessment Overview.docxAssessment Task Descriptions and Rubrics.docxAssessment Station Examples.mp4 [file mep_2374-8265.11405-s001.zip › E. Assessment Task Descriptions and Rubrics.docx]

| \| Laparoscopic Assessment \| 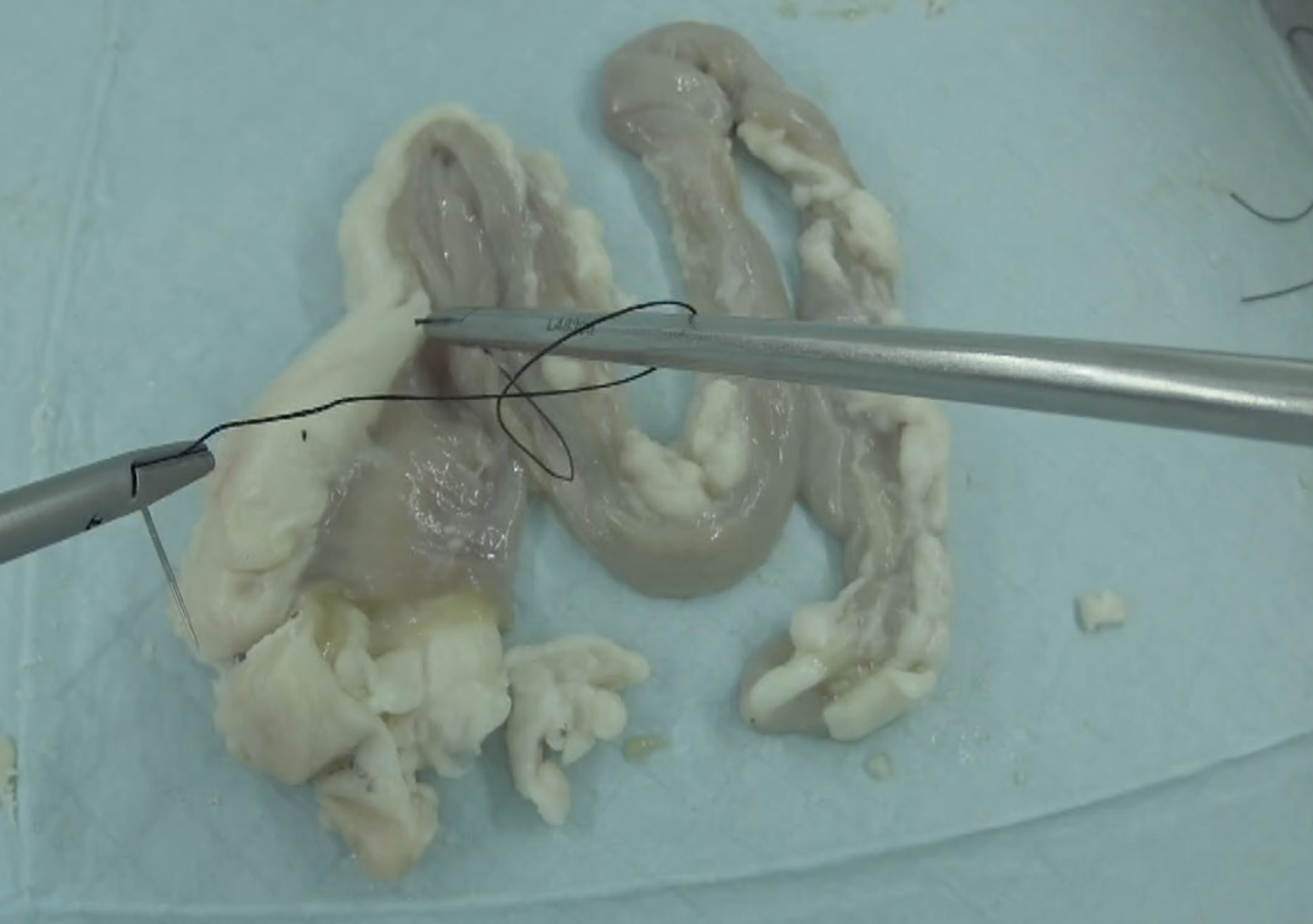 \| \| --- \| --- \| \| 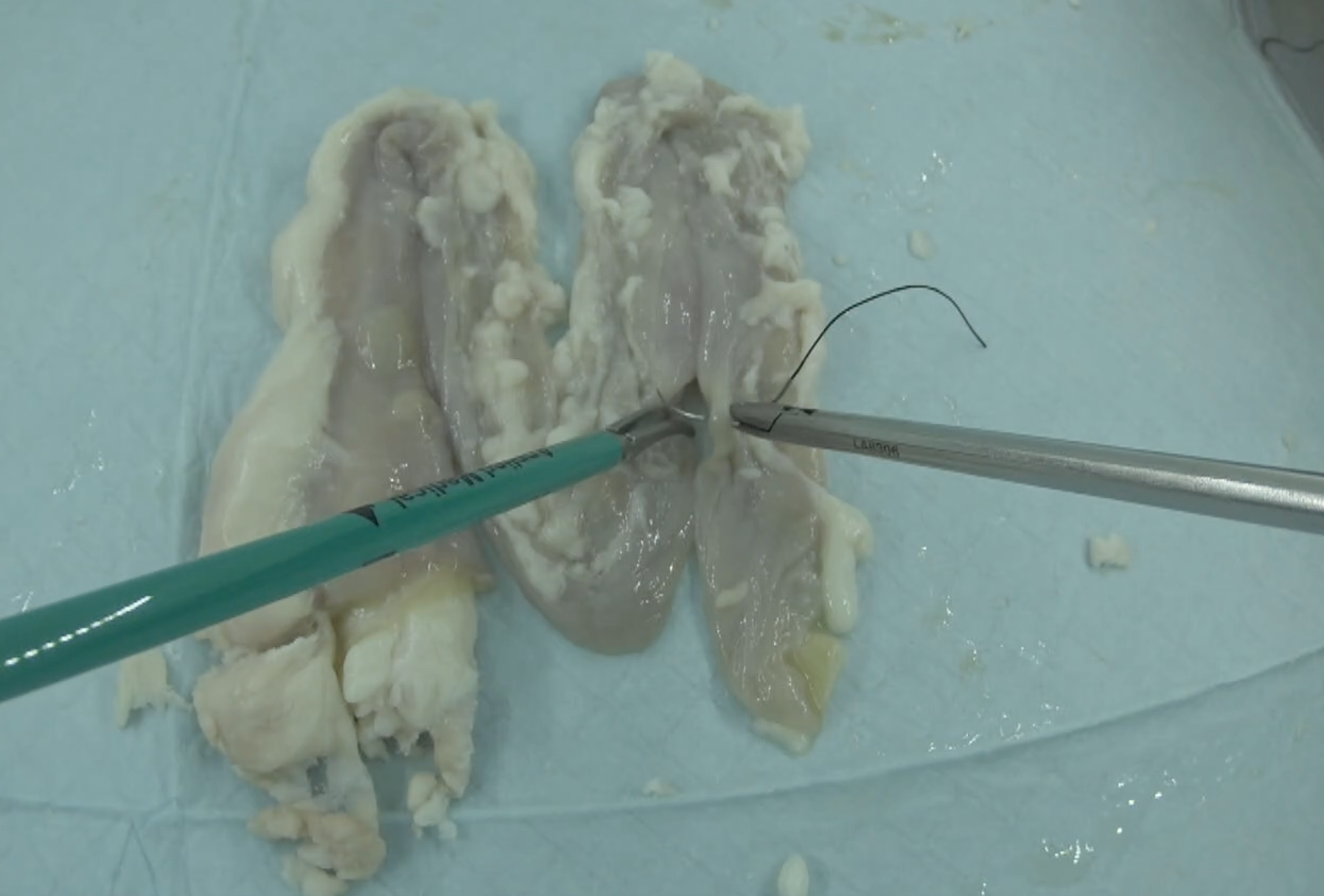 \| 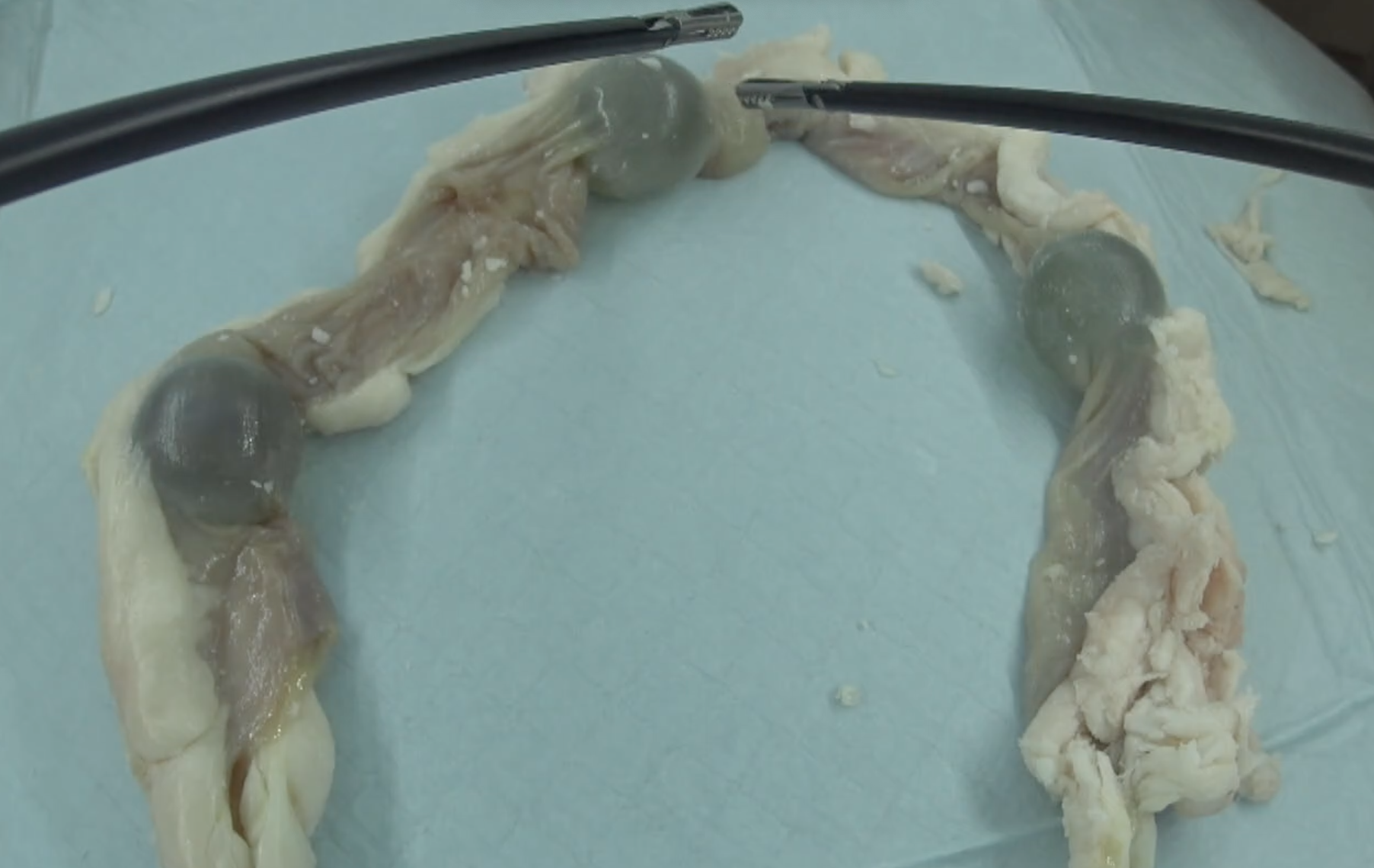 \| |
| --- | --- | --- | --- | --- |
| Assessment Task Descriptions and Rubrics  This manual includes information on performing and assessing the in-person laparoscopic tasks. Provide this manual to residents prior to starting the assessment and to faculty members during the assessment. The assessment can be performed during a one to two hour session.  Note that all pictures are author created and owned and have not been publicly distributed previously. |

## STATION ONE: ENTEROTOMY + FOREIGN BODY RETRIEVAL

*Task description:* Use a pair of scissors in your dominant hand and a grasper in your nondominant hand. Have a second grasper nearby. Identify a marble (“foreign body”) in the bowel. Grasp the bowel above or below the marble (not directly over the marble) with your grasper. Make an antimesenteric enterotomy in one cut with your dominant hand. Use two graspers to milk the marble out the enterotomy. The time starts when you first touch the bowel and ends when the marble has completely exited the bowel. Aim to complete the task in 55 seconds or less.

- Tension
  - **3 points:** Appropriate tension is maintained with the nondominant hand by grabbing and triangulating
  - **2 points:** Appropriate tension is occasionally or suboptimally maintained with the nondominant hand
  - **1 point:** The nondominant hand is not used to provide tension
- Precision
- **4 points:** An enterotomy is created in one cut on the antimesenteric side of the bowel
- **3 points:** An enterotomy is created on the antimesenteric side of the bowel in more than one cut
- **2 points:** An enterotomy is created but not on the antimesenteric side of the bowel
- **1 point:** No enterotomy is created
- Forward Planning
- **2 points:** The enterotomy is created in an appropriate location to allow for efficient foreign body retrieval
- **1 point:** The enterotomy does not optimize foreign body retrieval
- Instrumentation
- **2 points:** The instruments are manipulated smoothly to complete each part of the task
- **1 point:** The instruments are not manipulated smoothly to complete each part of the task
- Time
- **4 points:** 55 seconds or less
- **3 points:** 56 to 80 seconds
- **2 points:** 81 to 105 seconds
- **1 point:** 106 seconds or more

## STATION TWO: RUNNING THE BOWEL

*Task description:* Use two graspers. The bowel will be curled in the upper left aspect of the screen. Starting with the free end of the bowel, run the length of the bowel and search for enterotomies. When you see an enterotomy, state, “here is an enterotomy.” Do not pull the bowel out of the clip. The time starts when you first touch the bowel and ends when your grasper last releases the bowel. Aim to complete the task in 58 seconds or less.

- Visuospatial
- **3 points:** Both instruments are kept in the field of view
- **2 points:** An instrument is moved out of the field of view while the jaws are empty
- **1 point:** An instrument is moved out of the field of view while grasping the bowel
- Handling
- **3 points:** The bowel is not pulled out of or moved within the clip
- **2 points:** The bowel is moved within the clip but not pulled out
- **1 point:** The bowel is pulled out of the clip
- Instrumentation
- **2 points:** The instruments are manipulated smoothly to complete each part of the task
- **1 point:** The instruments are not manipulated smoothly to complete each part of the task
- Enterotomy
- **2 points:** An enterotomy is identified
- **1 point:** An enterotomy is not identified
- Time
- **4 points:** 58 seconds or less
- **3 points:** 59 to 78 seconds
- **2 points:** 79 to 98 seconds
- **1 point:** 99 seconds or more

## STATION THREE: NEEDLE LOADING

*Task description:* Use a needle driver in your dominant hand and a Maryland grasper or a second needle driver in your nondominant hand. Introduce a needle with at least a 5cm tail onto the field ***with your nondominant hand.*** Load the needle onto the needle driver in a forehand configuration. After loading the needle, drop the needle. Introduce a second needle with at least a 5cm tail onto the field with your ***dominant hand***. Use your nondominant hand to load the needle onto the needle driver in your dominant hand in a forehand configuration. The time starts when the first needle can be seen on the screen and ends when the second needle is loaded in the dominant hand. Aim to complete the task in 25 seconds or less.

- Instrumentation (1)
- **3 points:** The nondominant hand manipulates the needle to present it in the appropriate orientation for the needle driver without having to be reloaded
- **2 points:** The nondominant hand manipulates the needle to present it in the appropriate orientation for the needle driver, but requires one or more reloadings
- **1 point:** The nondominant hand does not present the needle in the appropriate orientation
- Instrumentation (2)
  - **2 points:** The instruments are manipulated smoothly to complete each part of the task
  - **1 point:** The instruments are not manipulated smoothly to complete each part of the task
- Dexterity
  - **3 points:** The needle is transferred from the nondominant hand to the needle driver and angled properly
  - **2 points:** The needle is transferred from the nondominant hand to the needle driver and angled in a suboptimal way
  - **1 point:** The needle is not transferred from the nondominant hand to the needle driver
- Time
  - **4 points:** 25 seconds or less
  - **3 points:** 26 to 36 seconds
  - **2 points:** 37 to 47 seconds
  - **1 point:** 48 seconds or more

## STATION FOUR: THROWING STITCHES

*Task description:* Use a needle driver in your dominant hand and a Maryland grasper or a second needle driver in your nondominant hand. Load a needle with a 5cm tail in your dominant hand before starting the task. Pass the needle through the antimesenteric side of two apposed segments of bowel. Repeat this twice for a total of three passes. No knot tying is required. The time starts when you first touch the bowel and ends when your grasper last releases the bowel after the third pass. Aim to complete the task in 75 seconds or less.

- Precision
- **4 points:** The needle passes through an appropriately sized bite on the antimesenteric sides of the apposed segments of bowel
- **3 points:** The needle passes through an inappropriately large or small bite on the antimesenteric sides of the apposed segments of bowel
- **2 points:** The needle passes through the apposed segments of bowel but not on the antimesenteric side of the bowel
- **1 point:** The needle does not pass through the bowel or tears the bowel
- Handling
- **2 points:** The bowel is not lifted off the tray
- **1 point:** The bowel is lifted off the tray

- Reloading
- **3 points:** The needle is reloaded between stitches with no wasted movements
- **2 points:** The needle is reloaded between stitches with some wasted movements
- **1 point:** The needle is dropped while reloading
- Tension
- **3 points:** Appropriate tension is maintained with the nondominant hand
- **2 points:** Appropriate tension is occasionally maintained with the nondominant hand
- **1 point:** The nondominant hand is not used to provide tension
- Instrumentation
- **2 points:** The instruments are manipulated smoothly to complete each part of the task
- **1 point:** The instruments are not manipulated smoothly to complete each part of the task
- Time
- **4 points:** 75 seconds or less
- **3 points:** 76 to 90 seconds
- **2 points:** 91 to 105 seconds
- **1 point:** 106 seconds or more

## STATION FIVE: SIMPLE INTERRUPTED EXTRACORPOREAL SUTURING

*Task description:* Use a needle driver in your dominant hand and a Maryland grasper or needle driver in your nondominant hand. Have scissors and a knot pusher nearby. Start with the needle loaded. Pass the needle through the antimesenteric side of two apposed segments of bowel. Use a suture of 90 to 120cm in length. Tie three single throws extracorporeally and secure them with the knot pusher. Cut the ends. The time starts when you first touch the bowel and ends when you cut the suture. Aim to complete the task in 99 seconds or less.

- Precision
- **4 points:** The needle passes through an appropriately sized bite on the antimesenteric sides of the apposed segments of bowel
- **3 points:** The needle passes through an inappropriately large or small bite on the antimesenteric sides of the apposed segments of bowel
- **2 points:** The needle passes through the apposed segments of bowel but not on the antimesenteric side of the bowel
- **1 point:** The needle does not pass through the bowel or tears the bowel
- Tension
- **3 points:** Appropriate tension is maintained with the nondominant hand
- **2 points:** Appropriate tension is occasionally maintained with the nondominant hand
- **1 point:** The nondominant hand is not used to provide tension
- Knot Security
- **3 points:** There is no air knot
- **2 points:** There is a small air knot, but the bowel is still well approximated
- **1 point:** There is a significant air knot and the bowel is not well approximated
- Handling
- **2 points:** The bowel is not lifted off the tray
- **1 point:** The bowel is lifted off the tray
- Instrumentation
- **2 points:** The instruments are manipulated smoothly to complete each part of the task
- **1 point:** The instruments are not manipulated smoothly to complete each part of the task
- Time
- **4 points:** 99 seconds or less
- **3 points:** 100 to 149 seconds
- **2 points:** 150 to 199 seconds
- **1 point:** 200 seconds or more

## STATION SIX: SIMPLE INTERRUPTED INTRACORPOREAL SUTURING

*Task description:* Use a needle driver in your dominant hand and a Maryland grasper or needle driver in your nondominant hand. Have scissors nearby. The suture will already be placed in the bowel prior to starting this task. Tie a surgeon’s knot followed by two additional throws. You must exchange hands between each throw. Cut the ends. The time starts when your first instrument is visualized on the screen and ends when you cut the suture. Aim to complete the task in 96 seconds or less.

- Tail Management
- **3 points:** The tail end is kept sufficiently short to facilitate knot tying
- **2 points:** The tail end is too long but the tie is completed without creating a bow
- **1 point:** The tail end is too long and a bow is creating when tying
- Loop Management
- **3 points:** The two instruments move synchronously to form the loop and grab the tail end of the suture
- **2 points:** A loop is formed but only one instrument moves to grab the tail
- **1 point:** No loop is formed
- Knot Security
- **3 points:** There is no air knot
- **2 points:** There is a small air knot, but the bowel is still well approximated
- **1 point:** There is a significant air knot and the bowel is not well approximated
- Handling
- **2 points:** The bowel is not lifted off the tray
- **1 point:** The bowel is lifted off the tray
- Instrumentation
- **2 points:** The instruments are manipulated smoothly to complete each part of the task
- **1 point:** The instruments are not manipulated smoothly to complete each part of the task
- Time
- **4 points:** 96 seconds or less
- **3 points:** 97 to 160 seconds
- **2 points:** 161 to 244 seconds
- **1 point:** 245 seconds or more
