# Supplementary material for: An At-Home Laparoscopic Curriculum for Junior Residents in Surgery, Obstetrics/Gynecology, and Urology
Source: MedEdPORTAL. 2024 May 24;20:11405. doi: 10.15766/mep_2374-8265.11405 (PMC11219092; doi:10.15766/mep_2374-8265.11405)
Supplement: Supplementary file 1 — At-Home Task Examples.mp4At-Home Task Descriptions and Rubrics.docxEquipment.docxEnd-of-Curriculum Assessment Overview.docxAssessment Task Descriptions and Rubrics.docxAssessment Station Examples.mp4 [file mep_2374-8265.11405-s001.zip › _Educational Summary Report_11405.pdf]

MedEdPORTAL® The AAMC Journal of Teaching and Learning Resources

Original Publication Open Access

An At-Home Laparoscopic Curriculum for Junior Residents in Surgery, Obstetrics/Gynecology, and Urology

Riley Brian, MD, MAEd*, David Bayne, MD, MPH, Traci Ito, MD, Jeannette Lager, MD, MPH, Anya Edwards, MD, MBA, Sandhya Kumar, MD, Ian Soriano, MD, Patricia O'Sullivan, EdD, Julian Varas, MD, MS, Hueylan Chern, MD

*Corresponding author: riley.brian@ucsf.edu

Abstract

Introduction: Laparoscopic surgery requires significant training, and prior studies have shown that surgical residents lack key laparoscopic skills. Many educators have implemented simulation curricula to improve laparoscopic training. Given limited time for dedicated, in-person simulation center practice, at-home training has emerged as a possible mechanism by which to expand training and promote practice. There remains a gap in published at-home laparoscopic curricula employing embedded feedback mechanisms. **Methods:** We developed a nine-task at-home laparoscopic curriculum and an end-of-curriculum assessment following Kern's six-step approach. We implemented the curriculum over 4 months with first- to third-year residents. **Results:** Of 47 invited residents from general surgery, obstetrics/gynecology, and urology, 37 (79%) participated in the at-home curriculum, and 25 (53%) participated in the end-of-curriculum assessment. Residents who participated in the at-home curriculum completed a median of six of nine tasks (interquartile range: 3-8). Twenty-two residents (47%) responded to a postcurriculum survey. Of these, 19 (86%) reported that their laparoscopic skills improved through completion of the curriculum, and the same 19 (86%) felt that the curriculum should be continued for future residents. Residents who completed more at-home curriculum tasks scored higher on the end-of-curriculum assessment ($p = .009$ with adjusted R^2 of .28) and performed assessment tasks in less time ($p = .004$ with adjusted R^2 of .28). **Discussion:** This learner-centered laparoscopic curriculum provides guiding examples, spaced practice, feedback, and graduated skill development to enable junior residents to improve their laparoscopic skills in a low-stakes, at-home environment.

Keywords
Laparoscopic Simulation, Home Practice, Asynchronous Practice, OB/GYN, Simulation, Surgery - General, Urology

Educational Objectives

By the end of this activity, learners will be able to:

1. Coordinate commonly used laparoscopic instruments with dominant and nondominant hands to transfer objects and tissue.
2. Provide retraction and tension with the nondominant hand to cut laparoscopically.
3. Perform laparoscopic suturing with intracorporeal and extracorporeal knot tying to place secure stitches.

Citation:
Brian R, Bayne D, Ito T, et al. An at-home laparoscopic curriculum for junior residents in surgery, obstetrics/gynecology, and urology. *MedEdPORTAL*. 2024;20:11405.
https://doi.org/10.15766/mep_2374-8265.11405

Introduction

Compared with traditional open surgery, laparoscopic surgery is associated with reduced pain and shortened length of hospital stay.[1-3] Unfortunately, acquiring the skills to perform laparoscopic surgery is challenging, and prior work has demonstrated that a significant proportion of senior surgical trainees struggle to perform laparoscopic procedures.[4-6] To address this problem, many educators have turned to laparoscopic simulation, which can improve trainee performance on simulated tasks and in the operating room.[7-10] In fact, prior work has suggested that laparoscopic simulation may enable trainees to outperform attending surgeons.[11]

Simulation curricula, when based on the theory of deliberate practice, require trainees to practice and receive feedback for optimal efficacy.[12] However, trainees and trainers have limited dedicated time for in-person simulation center practice and feedback.[13] To increase practice, trainees can spend time at home, away from the simulation center.[14] Remote and virtual

Copyright © 2024 Brian et al. This is an open-access publication distributed under the terms of the Creative Commons Attribution license.

learning became widespread during the coronavirus pandemic, with ongoing applications for ease and learner preference.[15-19] Off-site training with remote, asynchronous feedback is a means by which trainees can practice skills at an individualized pace from a convenient location.[20,21]

Previous work has described the general feasibility and benefits of at-home laparoscopic practice. Several prior curricula have shown at-home practice to be easily used and efficacious for skill acquisition.[17,18,22,23] These curricula have employed quite varied commercial and noncommercial trainers with diverse training paradigms.[7,13] Unfortunately, there are barriers to successful at-home laparoscopic simulation. A qualitative study previously reported that suboptimal feedback, particularly with regard to a lack of individualized feedback, prevented trainees from progressing through at-home practice.[14] That study also noted poor faculty engagement with the training curriculum. No publication currently exists in *MedEdPORTAL* that equips educators with the tools to implement at-home laparoscopic training. Thus, there remains a gap in published at-home laparoscopic training curricula with robust feedback mechanisms that provide sufficient information to guide trainee improvement.

To address this gap, we created an at-home learner-centered laparoscopic curriculum with an embedded feedback process and an end-of-curriculum assessment for junior residents in general surgery, obstetrics/gynecology, and urology. The curriculum aimed to facilitate laparoscopic skill development in a low-stakes at-home environment.

Methods

Development

We developed a nine-task at-home laparoscopic curriculum and an end-of-curriculum assessment following Kern's six-step approach.[24] We began with problem identification and a needs assessment to determine deficiencies in laparoscopic training. We conducted the needs assessment through in-depth interviews with 14 laparoscopic surgeons in general surgery, obstetrics/gynecology, and urology.[25] Results from the needs assessment informed the goals, objectives, and educational strategies for the curriculum. We derived at-home curricular tasks from previously published in-person curricula, including the Fundamentals of Laparoscopic Surgery (FLS)[26] and Lapp.[27] The nine tasks were peg transfer, running the bowel, pattern cutting, needle loading (part 1), needle loading (part 2), interrupted extracorporeal suturing, interrupted intracorporeal suturing (part 1), running suturing, and interrupted intracorporeal suturing (part 2; Appendix A). We designed the curricular flow so that tasks built on previously completed ones; for example, the needle loading tasks preceded basic suturing tasks, which preceded complex suturing tasks.

We created assessment tools for the at-home curriculum and end-of-curriculum assessment by following a standard process for applied measurement tool development, which involved defining the relevant constructs around laparoscopic ability, designing items related to the construct, and determining possible outcomes for each item.[28] We aimed to create formative items and outcomes. More specifically, we designed items and outcomes so that residents could read the items and outcomes and then know how to improve their performance. We incorporated time suggestions for at-home practice from gold-standard recommendations for previously published tasks.[29] We refined items and their possible outcomes on the scoring rubrics by determining content and response process validity through discussion with expert laparoscopic surgeons, educators, and residents. The resulting task descriptions and rubrics are available in Appendix B.

Equipment/Environment

We assembled equipment for the at-home curriculum and the end-of-curriculum assessment (Appendix C). Except for laparoscopic knot pushers, ribbon, and bovine intestine, we obtained materials through donation.

We distributed at-home curriculum equipment to each resident. Resident participants then conducted the tasks for the at-home curriculum asynchronously in locations of their choosing. We conducted the end-of-curriculum assessment in a central surgical skills center with laparoscopic trainers.

Personnel

Key personnel who implemented this curriculum included a staff member at the skills center with experience preparing surgical simulation sessions and faculty members with laparoscopic experience.

The staff member at the skills center helped assemble and distribute the materials for the at-home laparoscopic curriculum described above. The staff member also helped residents follow the task completion timeline. The faculty members provided feedback for at-home tasks and facilitated the end-of-curriculum assessment, further described below.

Implementation

We implemented the curriculum among first- to third-year residents in three residency programs at our institution: general

Copyright © 2024 Brian et al. This is an open-access publication distributed under the terms of the Creative Commons Attribution license.

surgery, obstetrics/gynecology, and urology. We began by introducing the curriculum to residents at the end of an in-person simulation session in which we discussed laparoscopic surgery, basic laparoscopic instruments, and key differences between laparoscopic and open techniques. We provided residents with time during this introductory session to practice with laparoscopic instruments on the FLS tasks. Residents could proceed in the curriculum even if they missed this session. We subsequently emailed residents details including the task examples (Appendix A), task descriptions (Appendix B), and expected timeline for task completion. We asked residents to complete one video of a task every 2 weeks (the timeline could be adjusted as needed). We did not require that the videos meet any quality standards; we conveyed that residents could submit their first video or practice as much as they liked before choosing a video to submit. This flexibility permitted residents to focus on practicing skills when their schedules and duty hours permitted. We requested that residents submit videos online, though compressed videos could also be emailed to assessors. We emailed residents prior to each completion deadline. We also sent reminder emails when residents did not submit videos on time.

Following video submission, faculty members and residents reviewed the videos and gave asynchronous feedback to residents about task performance using the rubrics and additional free-form written comments. We had previously provided faculty members with the feedback rubrics and reviewed examples of different outcomes with them. We asked participating residents to review their feedback for each task before continuing to the next task.

Assessment

After residents had completed the nine tasks in the at-home curriculum, we invited them to an in-person, end-of-curriculum assessment to transfer their laparoscopic skills from the at-home curriculum to a tissue model, obtain additional feedback, appreciate their skill progression, and demonstrate confidence as they prepared for laparoscopic surgery in the operating room (Appendix D).

We created rubrics with items and outcome spaces analogous to the at-home laparoscopic tasks, for which we had previously developed content and response process validity. We provided residents with the details and rubrics for the end-of-curriculum assessment (Appendix E) as well as example videos (Appendix F).

During the session, assessors filled out the assessment rubrics but also focused on providing ongoing feedback to residents to optimize laparoscopic ability. Following the assessment session, we conducted two linear regressions: one to assess the relationship between the number of at-home curriculum tasks completed by a resident and the resident's performance on the end-of-curriculum assessment and a second to assess the relationship between the number of at-home curriculum tasks completed by a resident and the resident's time to complete assessment tasks. We used chi-square tests to determine differences in curricular participation based on specialty and training level. We performed analyses in Stata/IC version 16.1 for Mac (StataCorp).

Debriefing

We debriefed in person after residents had completed the tasks from the end-of-curriculum assessment. We gathered both residents and faculty assessors to discuss how at-home practice translated to operating on tissue, barriers to performance and tips on overcoming them, key strategies for challenging skills such as loop formation and tail management, and the importance of ongoing skill development as well as methods to continue asynchronous at-home practice (Appendix D). We also distributed a postcurriculum survey to all residents to allow for curricular feedback.

All pictures and videos in the aforementioned appendices (Appendices A-F) are author created and owned and have not been publicly distributed previously. Our institutional review board exempted this curricular evaluation (UCSF IRB 21-33846, 2021).

Results

We invited 47 junior residents from general surgery, obstetrics/gynecology, and urology to participate, of whom 37 (79%) participated in the at-home curriculum and 25 (53%) in the end-of-curriculum assessment. We conducted process, impact, and effectiveness evaluations.

Residents who participated in the at-home curriculum completed a median of six of nine tasks (interquartile range [IQR]: 3-8) and spent a median of 60 minutes (IQR: 45-68) practicing, recording, and uploading each task. Curricular participation did not significantly differ based on specialty ($p = .14$) or training level ($p = .76$).

Twenty-two residents (47%) responded to the postcurriculum survey. These residents' laparoscopic experience ranged substantially, though most stated that they had operated laparoscopically in one to four prior cases ($n = 9$). Among survey respondents, 19 (86%) reported that their laparoscopic

Copyright © 2024 Brian et al. This is an open-access publication distributed under the terms of the Creative Commons Attribution license.

skills improved through completion of the curriculum, and the same 19 (86%) felt that the curriculum should be continued for future residents. Of the three residents who reported that their skills did not improve, two noted in free response that they preferred in-person skills sessions. Sixteen of 21 residents (76%) found the grading rubrics moderately or very helpful in completing the tasks, and 18 of 19 residents (95%) agreed that they knew how to improve their performance after their tasks were graded. Several residents noted equipment challenges in free-text responses.

We evaluated the effectiveness of the at-home curriculum by performing an end-of-curriculum assessment and analyzing the association between at-home curriculum task completion and assessment performance. We found that residents who completed more at-home curriculum tasks scored higher on the end-of-curriculum assessment ($p = .009$ with adjusted R^2 of .28; Figure 1). We also found that residents who completed more at-home curriculum tasks performed tasks in less time on the end-of-curriculum assessment ($p = .004$ with adjusted R^2 of .28; Figure 2).

Discussion

Here, we have described a novel at-home laparoscopic curriculum and end-of-curriculum assessment for junior residents in surgery, obstetrics/gynecology, and urology. The tasks in the curriculum allow residents to learn laparoscopic instrument coordination, retraction, tension, suturing, and knot tying. Residents who completed more tasks in the at-home curriculum scored better during the end-of-curriculum assessment, which measured the Educational Objectives. The curriculum is easy to implement and has been well received.

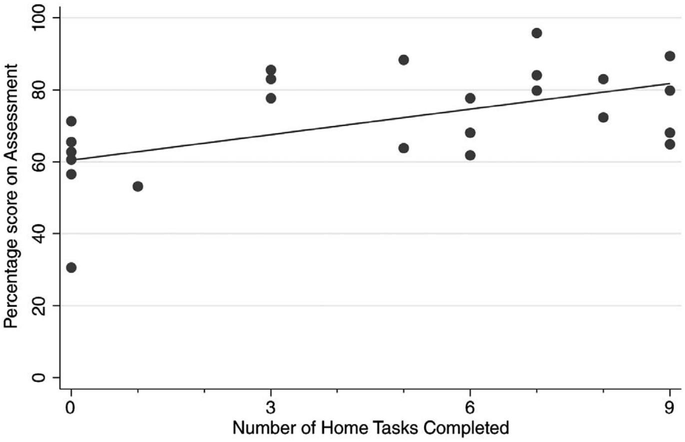

Figure 1. Residents who completed more tasks from the home curriculum had higher scores on the end-of-curriculum assessment ($p = .009$ with adjusted $R^2 = .28$).

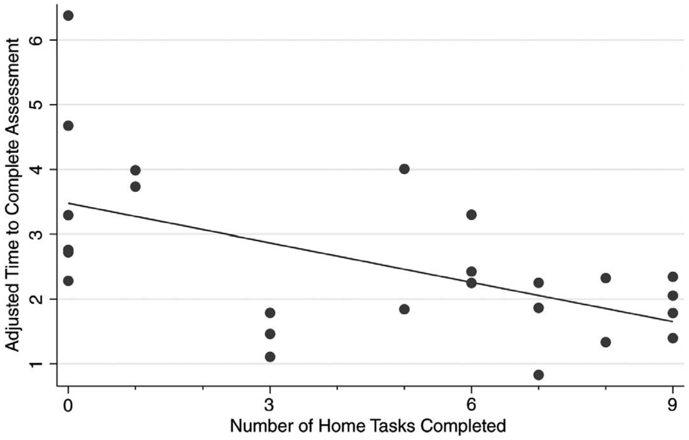

Figure 2. Residents who completed more tasks from the home curriculum completed all tasks more quickly on the end-of-curriculum assessment ($p = .004$ with adjusted $R^2 = .28$).

Given the learning curve associated with performing laparoscopic surgery, multiple prior studies have evaluated a number of methods to optimize resident learning.[4] Indeed, asynchronous simulation with resident practice and video recordings in simulation centers have shown promise in increasing access to surgical simulation.[30] Similarly, remote simulation has gained traction, particularly with the coronavirus pandemic.[19] Our curriculum builds on prior reports by incorporating several previously published tasks, time guidelines, and implementation strategies.[13,14,29] In addition, it expands on prior work by using the theory of deliberate practice to outline a step-by-step at-home laparoscopic curriculum with guiding examples, spaced practice, engaged faculty feedback, and graduated skill development. The materials used to implement the curriculum are readily available and can be harnessed by training programs to expand surgical simulation in an asynchronous format.

There are multiple limitations to this curriculum. We implemented it at a single large academic medical center for general surgery, obstetrics/gynecology, and urology junior residents. The curriculum is most likely to be successful at similar academic residency training programs with laparoscopic surgeons to help drive implementation and feedback. Specific programs also need to determine whether schedules and duty hours permit at-home practice. In addition, not all invited residents participated in the curriculum, the end-of-curriculum assessment, or the survey. Residents who did not participate in one or more

Copyright © 2024 Brian et al. This is an open-access publication distributed under the terms of the Creative Commons Attribution license.

aspects frequently cited limited time as a barrier to greater involvement, which has previously been suggested to be a limitation to trainees' at-home simulation.[14] Nonetheless, this may have biased results if residents with greater interest or ability in laparoscopy constituted more of the participating population. The number of participants limited our ability to assess performance based on covariates other than at-home curricular participation. Finally, the curriculum employed newly created rubrics for task assessment. While we followed a rigorous process in creating these instruments and assessed several strands of validity, additional validity evidence would better support the rubrics' widespread use in assessment. Notably, we developed the rubrics with a major goal of providing formative feedback through their completion.

Residents provided concrete suggestions for curricular improvement. Specifically, some noted challenges in setting up box trainers. We have updated the latest iteration of our curriculum to address these concerns by incorporating a step-by-step guide for box trainer setup. Others implementing the curriculum may need to guide residents depending on the specific equipment used.

Overall, we have outlined an at-home learner-centered laparoscopic curriculum and an end-of-curriculum assessment for junior residents in general surgery, obstetrics/gynecology, and urology. The curriculum facilitates laparoscopic skill development in a low-stakes at-home environment.

Appendices

A. At-Home Task Examples.mp4

B. At-Home Task Descriptions and Rubrics.docx

C. Equipment.docx

D. End-of-Curriculum Assessment Overview.docx

E. Assessment Task Descriptions and Rubrics.docx

F. Assessment Station Examples.mp4

All appendices are peer reviewed as integral parts of the Original Publication.

Riley Brian, MD, MAEd: Research Resident, Department of Surgery, University of California, San Francisco; ORCID: https://orcid.org/0000-0002-7462-3214

David Bayne, MD, MPH: Assistant Professor, Department of Urology, University of California, San Francisco

Traci Ito, MD: Assistant Professor, Department of Obstetrics, Gynecology & Reproductive Sciences, University of California, San Francisco

Jeannette Lager, MD, MPH: Professor, Department of Obstetrics, Gynecology & Reproductive Sciences, University of California, San Francisco

Anya Edwards, MD, MBA: First-Year Resident, Department of Surgery, University of California, San Francisco

Sandhya Kumar, MD: Assistant Professor, Department of Surgery, University of California, San Francisco

Ian Soriano, MD: Associate Professor, Department of Surgery, University of California, San Francisco

Patricia O'Sullivan, EdD: Professor, School of Medicine, University of California, San Francisco

Julian Varas, MD, MS: Associate Professor, Surgical Division, Faculty of Medicine, Pontificia Universidad Católica de Chile

Hueylan Chern, MD: Professor, Department of Surgery, University of California, San Francisco

Disclosures
None to report.

Funding/Support
Dr. David Bayne received funding from the University of California, San Francisco, Academy of Medical Educators as Innovations Funding 2022-2023.

Dr. Riley Brian received funding from Intuitive Surgical as an Intuitive Surgical-University of California, San Francisco, Simulation-Based Surgical Education Research Fellowship.

Ethical Approval
The University of California, San Francisco, Institutional Review Board deemed further review of this project not necessary.

References

1. Arezzo A. The past, the present, and the future of minimally invasive therapy in laparoscopic surgery: a review and speculative outlook. *Minim Invasive Ther Allied Technol*. 2014; 23(5):253-260. https://doi.org/10.3109/13645706.2014.900084
2. Bates AT, Divino C. Laparoscopic surgery in the elderly: a review of the literature. *Aging Dis*. 2015;6(2):149-155. https://doi.org/10.14336/AD.2014.0429
3. Zhang FW, Zhou ZY, Wang HL, et al. Laparoscopic versus open surgery for rectal cancer: a systematic review and meta-analysis of randomized controlled trials. *Asian Pac J Cancer Prev*. 2014; 15(22):9985-9996. https://doi.org/10.7314/APJCP.2014.15.22.9985
4. Brian R, Davis G, Park KM, Alseidi A. Evolution of laparoscopic education and the laparoscopic learning curve: a review of the

Copyright © 2024 Brian et al. This is an open-access publication distributed under the terms of the Creative Commons Attribution license.

literature. *Laparosc Surg*. 2022;6:34. https://doi.org/10.21037/ls-22-29

5. George BC, Bohnen JD, Williams RG, et al; Procedural Learning and Safety Collaborative (PLSC). Readiness of US general surgery residents for independent practice. *Ann Surg*. 2017;266(4): 582-594. https://doi.org/10.1097/SLA.0000000000002414
6. Bohnen JD, George BC, Zwischenberger JB, et al. Trainee autonomy in minimally invasive general surgery in the United States: establishing a national benchmark. *J Surg Educ*. 2020; 77(6):e52-e62. https://doi.org/10.1016/j.jsurg.2020.07.033
7. Li MM, George J. A systematic review of low-cost laparoscopic simulators. *Surg Endosc*. 2017;31(1):38-48. https://doi.org/10.1007/s00464-016-4953-3
8. Nepomnayshy D, Alseidi AA, Fitzgibbons SC, Stefanidis D. Identifying the need for and content of an advanced laparoscopic skills curriculum: results of a national survey. *Am J Surg*. 2016;211(2):421-425. https://doi.org/10.1016/j.amjsurg.2015.10.009
9. Zendejas B, Brydges R, Hamstra SJ, Cook DA. State of the evidence on simulation-based training for laparoscopic surgery: a systematic review. *Ann Surg*. 2013;257(4):586-593. https://doi.org/10.1097/SLA.0b013e318288c40b
10. Palter VN, Grantcharov TP. Development and validation of a comprehensive curriculum to teach an advanced minimally invasive procedure: a randomized controlled trial. *Ann Surg*. 2012;256(1):25-32. https://doi.org/10.1097/SLA.0b013e318258f5aa
11. Boza C, León F, Buckel E, et al. Simulation-trained junior residents perform better than general surgeons on advanced laparoscopic cases. *Surg Endosc*. 2017;31(1):135-141. https://doi.org/10.1007/s00464-016-4942-6
12. Anders Ericsson K. Deliberate practice and acquisition of expert performance: a general overview. *Acad Emerg Med*. 2008;15(11):988-994. https://doi.org/10.1111/j.1553-2712.2008.00227.x
13. Thinggaard E, Kleif J, Bjerrum F, et al. Off-site training of laparoscopic skills, a scoping review using a thematic analysis. *Surg Endosc*. 2016;30(11):4733-4741. https://doi.org/10.1007/s00464-016-4834-9
14. Blackhall VI, Cleland J, Wilson P, Moug SJ, Walker KG. Barriers and facilitators to deliberate practice using take-home laparoscopic simulators. *Surg Endosc*. 2019;33(9):2951-2959. https://doi.org/10.1007/s00464-018-6599-9
15. Dickinson KJ, Gronseth SL. Application of universal design for learning (UDL) principles to surgical education during the COVID-19 pandemic. *J Surg Educ*. 2020;77(5):1008-1012. https://doi.org/10.1016/j.jsurg.2020.06.005
16. Sabharwal S, Ficke JR, LaPorte DM. How we do it: modified residency programming and adoption of remote didactic curriculum during the COVID-19 pandemic. *J Surg Educ*. 2020;77(5):1033-1036. https://doi.org/10.1016/j.jsurg.2020.05.026
17. Joosten M, Bökkerink GMJ, Verhoeven BH, Botden SMBI. Evaluating the use of a take-home minimally invasive surgery box training for at-home training sessions before and during the COVID pandemic. *J Laparoendosc Adv Surg Tech A*. 2023;33(1):63-68. https://doi.org/10.1089/lap.2022.0197
18. Joosten M, Hillemans V, Bökkerink GMJ, de Blaauw I, Verhoeven BH, Botden SMBI. The feasibility and benefit of unsupervised at-home training of minimally invasive surgical skills. *Surg Endosc*. 2023;37(1):180-188. https://doi.org/10.1007/s00464-022-09424-2
19. Park KM, Rashidian N, Anderson C, et al. Evidence-based guidelines on the use of virtual surgical education pertaining to the domains of cognition and curriculum, psychomotor skills training, and faculty development and mentorship. *Ann Surg*. 2022;276(1):e6-e15. https://doi.org/10.1097/SLA.0000000000005014
20. Jarry Trujillo C, Achurra Tirado P, Escalona Vivas G, Crovari Eulufi F, Varas Cohen J. Surgical training during COVID-19: a validated solution to keep on practicing. *Br J Surg*. 2020;107(11): e468-e469. https://doi.org/10.1002/bjs.11923
21. Quezada J, Achurra P, Jarry C, et al. Minimally invasive tele-mentoring opportunity—the Mito Project. *Surg Endosc*. 2020;34(6):2585-2592. https://doi.org/10.1007/s00464-019-07024-1
22. Thinggaard E. Take-home training in laparoscopy. *Dan Med J*. 2017;64(4):B5335.
23. Nagaraj MB, AbdelFattah KR, Scott DJ, Farr DE. Creating a proficiency-based remote laparoscopic skills curriculum for the COVID-19 era. *J Surg Educ*. 2022;79(1):229-236. https://doi.org/10.1016/j.jsurg.2021.06.020
24. Thomas PA, Kern DE, Hughes MT, Chen BY, eds. *Curriculum Development for Medical Education: A Six-Step Approach*. 3rd ed. Johns Hopkins University Press; 2016.
25. Charondo LB, Brian R, Syed S, et al. Confronting new challenges: faculty perceptions of gaps in current laparoscopic curricula in a changing training landscape. *Surg Open Sci*. 2023;16:1-7. https://doi.org/10.1016/j.sopen.2023.09.006
26. Ritter EM, Scott DJ. Design of a proficiency-based skills training curriculum for the Fundamentals of Laparoscopic Surgery. *Surg Innov*. 2007;14(2):107-112. https://doi.org/10.1177/1553350607302329
27. Ulloa G, Neyem A, Escalona G, Ortiz C, Varas J. Remote asynchronous feedback for unsupervised laparoscopic training: the “Lapp” platform. *Arq Bras Cir Dig*. 2023;35:e1712. https://doi.org/10.1590/0102-672020220002e1712
28. Wilson M, Sloane K. From principles to practice: an embedded assessment system. *Appl Meas Educ*. 2000;13(2):181-208. https://doi.org/10.1207/S15324818AME1302_4

Copyright © 2024 Brian et al. This is an open-access publication distributed under the terms of the Creative Commons Attribution license.

29. Cassera MA, Zheng B, Swanström LL. Data-based self-study guidelines for the Fundamentals of Laparoscopic Surgery examination. *Surg Endosc*. 2012;26(12):3426-3429. https://doi.org/10.1007/s00464-012-2357-6

30. Gaete MI, Belmar F, Cortés M, et al. Remote and asynchronous training network: from a SAGES grant to an eight-country remote laparoscopic simulation training program. *Surg Endosc*. 2023; 37(2):1458-1465. https://doi.org/10.1007/s00464-022-09386-5

Received: September 1, 2023
Accepted: February 27, 2024
Published: May 24, 2024

Copyright © 2024 Brian et al. This is an open-access publication distributed under the terms of the Creative Commons Attribution license.
